# Notes and News

**Published:** 1889-11-02

**Authors:** 


### NOTES AND NEWS.

COLLECTED AND COMMUNICATED.

MR. AUGUSTUS J. HARVEY has made a discovery which will be the annihilation of the medical profession; he says a dose of thought will cure any disease. But are there not certain persons in whom the thinking apparatus is wanting One would naturally conclude so after perusing Mr. Harvey's printed slips.

THE "Waifs and Strays" Society have just issued their quarterly statement. There are at present 1,390 children under the care of the society, 683 being in the homes, and 428 boarded out; whilst there are 279 in other Church homes, for whose maintenance the society is responsible. The total number of children provided with homes since the society was founded nine years ago is 2,157.

THE Lady Superintendent of Torbay Hospital writes to explain the paragraph in their annual report, to which we referred on Oct. 12th. The late Mr. Pepprell, for many years secretary to the hospital, had a great deal to do with public monies; he went on a voyage to Australia, and after he left serious defalcations were discovered; but with regard to the hospital accounts all was in perfect order. Hence the surprise expressed by the committee in their report.

THE Mont Dore cure for which consumptives used to travel to Auvergnes has now been carried on for some three years at Bournemouth, and Mr. George Mahomed has written a pamphlet on some of the cases treated there. The cure chiefly consists in hot-air, hot-steam, and hot-water baths, and sea-water or the products of the pine can be applied with them. The cases quoted in the pamphlet are perhaps not quite satisfactory, nor quite numerous enough; but it is early days yet to demand a full account of what is a comparatively new treatment for many diseases.

THE relation between morality and cleanliness is pretty close we all know, but the Rev. Dr. Crosby is too thorough a Yankee to admit that the bad paving of New York makes it inferior as a city to anything in the old country. He says: "New York compares favourably with all the great cities of the world. In point of morality as well as in physical cleanliness the city is, on the whole, very far superior to the cities of the Continent of Europe, and superior even to the cities of Great Britain. Recently I have seen much of Liverpool and Glasgow. In point of public morality and sobriety, and the safety of people on the streets, they do not begin to compare with New York." This is rather amusing to those who know the disgracefully dirty streets of New York, and that there are also houses not of the highest character.

ONE story always calls forth another. A correspondent commenting on our tale about the temperance lecturer tells of a cobbler who, on being asked to drink water, remarked, "Water! why look how it rots the soles of your boots, and they're leather. Why what must it do to the coats of your stomach?"

A PERFECT epidemic of suicide seems to have attacked the medical profession, and has now spread from London to New York. Quite lately Dr. Hugh Sutherland, of East Fourteenth-street, took morphine, and though he was removed to New York Hospital and his colleagues did their best for him, he died next morning.

THE anti-vaccinators must be jubilant, for they have at last got a case which will no doubt be made the most of. The case was reported to the Bromley Guardians last Friday. A child named Grace Edith Buckwell was vaccinated by the medical officer on Sept. 10, and appeared to be going on satisfactorily, but unfavourable symptoms set in, and the child subsequently died. The medical officer, Dr. Codd, reported that the child died from lockjaw which followed up and was due to vaccination. He had never before seen a death or even serious illness resulting from vaccination, and its present occurrence he regarded as phenomenal.

WHEN the Darlington Hospital was built, about five years ago, the committee were unable to complete the original plans owing to lack of funds for the purpose. The Misses Pease, of Southend, Darlington, have now generously placed in the hands of the committee the sum of £500 for building a set of rooms and offices which, by providing for the servants, will free the rooms originally intended for the use of the nurses. One very interesting feature with regard to Darlington Hospital is the systematic manner in which the workmen of the town contribute to the funds. Some time ago now, a sad disaster brought to the hospital fifteen very serious cases within an hour, and all were treated with skill at once.

THE Devon and Cornwall Ear and Throat Hospital had 321 cases under treatment last year, of which 46 were re-entries, leaving 275 new cases, as against 246 in the previous year. Of those 81 were cured, 158 much relieved, 63 lost sight of, 17 incurable, and two had died. Cases classified as lost sight of were those who had attended once or twice and then disappeared, without affording sufficient opportunity of trying treatment. Compared with the previous year, a much greater number was relieved, though not quite so many were cured. Mr. H. B. Mildmay has just been elected president of this institution, and it is hoped that during his reign some beds may be provided for the treatment of the worst cases as in-patients.

THE quarterly meeting was held at Leicester Infirmary on Oct. 8th. Mr. T. F. Johnson gave the customary report on the state of the funds at the close of the last quarter. On the medical library account there was a balance in hand of £22 2s. 6d., on the fever house account a favourable balance of £74 18s. 3d., and on the chaplain's fund account a sum of £63 16s. 11d. in hand. On the infirmary account the balance was on the other side, there being £755 3s. 2d. due to the treasurer. Having referred to the great loss sustained by the infirmary in the death of Dr. Blunt, Mr. Johnson announced that the Children's Hospital was now open. The children had been transferred there. They looked very comfortable, and he hoped would be benefited by the change. They had at the infirmary the advantage of gentlemen coming there as visitors, and during the past few weeks they had as visitors four—Messrs. Franks, Holmes, Inskip, and Kell—distinctly representing the working classes. They had made an exhaustive examination of the state of things in the infirmary, and their report was thoroughly satisfactory.

The following vacancies are announced this week, the amount of the salary in each case being given after the name of the institution :

**Resident Medical Officers.**

South-Western Fever Hospital. Two Clinical Assistants—Board, &c.
North-West London Hospital. Junior Medical Officer.
City of London Hospital. Medical Officer—£100, board, &c.
Salop Infirmary. House Surgeon.—£100, board, &c.
City of London Hospital. House Physician—Board, &c.
Leicester Infirmary. Assistant House Surgeon—£80, board, &c.
Central London Sick Asylum. Assistant Medical Officer and Dispenser—£100, board, &c.

**Non-Resident Medical Officers.**

Borough of Huddersfield. Medical Officer of Health—£400.
Charing Cross Hospital. Surgical Registrar—£40.
Victoria Hospital for Children. Ophthalmic Surgeon.
Liverpool Royal Infirmary. Honorary Surgeon.
Parish of Eday, Orkney. Medical Officer—£70.
Gordon Hospital for Fistula. Assistant Surgeon.
Middlesex Hospital. Second Chloroformist.
Salisbury Infirmary. Dispenser—£105.

---

Dr. Magnan has read a paper before the Biological Society of Paris on insanity among anti-vivisectionists, a complaint he avers to be very common. One anti-vivisectionist dame he knew directed her energies towards cats, and had a veritable "hospital," with beds, blankets, and savoury food, for sick "Tabbies." Among the anti-vivisectionists, too, was a lady who became a vegetarian, in order not to eat the flesh of animals. Her hobby was to go every morning to the slaughter-yard at Villette in order to abuse roundly the slayers of oxen.

---

On October 24th, a large meeting was held by the Balloon Society, and Mr. Frank Kerslake delivered a lecture in favour of the compulsory muzzling of dogs. Mr. Kerslake gave harrowing particulars of deaths from hydrophobia, which he said had numbered 935 in the last 38 years. Mr. Brudenell Carter wrote, "It is a disgrace to humanity that mankind should be still exposed to the most horrible of all maladies when the means of remedy are so easy and so safe." Mr. Smith-Wright, M.P., Sir Wm. Moore, surgeon-general in India, Dr. Drysdale, and Mr. Lefevre took part in the proceedings.

---

It seems impossible to follow the quarrel at the Suffolk General Hospital. The discussions are so long, the personalities so frequent, that one drops the whole subject with sadness. To call the governors "dummies" is a sally greeted with loud laughter, and, indeed, the actors in the quarrel seem to find much more amusement in it than we can. The following resolution, moved by the Rev. Harry Jones, has been passed by 31 to 9: "That the resignation of the medical officers be not accepted, on the ground that the proceedings at the meeting on September 17, which have caused them to tender their resignation, were inconsistent with the unanimous decision of the General Board on June 18."

---

A correspondent of one of the evening papers declares he cannot prosper in the medical profession because he has too little money and too much hair. We have nothing to say about the little money, but as for the much hair it is an old story long since disproved. Perhaps at one time a bald head was regarded as suitable for the family medical attendant, but in these days the young doctor is as much in request as the old. We no longer lay such stress on experience; we appreciate rather the student fresh from his studies, and familiar with the latest triumphs of surgery. That man is indeed "a failure" who can give no better reason for too few patients than that he has too many hairs!

A mysterious disease is reported from Whitehall, Illinois, which goes by the name of bloody flux. During August there were 31 deaths in the city, of which 27 were children. The disease was also epidemic at Warsaw and other towns. It seems to have been worthy of its awful name.

---

A disgraceful state of affairs has been discovered at the Rochford Hospital for Infectious Diseases, to which Southend patients are sent. A sub-committee of the Southend Local Board reported that the premises were not suitable for the purpose of a hospital, that they were old and badly constructed, that the staff of attendants was quite inadequate, that the furniture was neither sufficient nor clean, that there was an offensive smell in the yard caused by improper sanitary arrangements, and much more. The report also recommended that a series of improvements should be carried out at once. It is extraordinary what scandals can exist in our midst only to be discovered at last by chance. Well-managed hospitals are often grumbled at and complaints chronicled, yet a place without any drainage or ventilation, and wretchedly cold and uncomfortable, pursues the tenor of its way for years.

---

A very sad death has occurred at Vienna from glanders. Dr. Hoffman procured from Dr. Rowalski the bacillus of glanders. All the animals he injected with the bacillæ died of the horrible malady. At the beginning of October Dr. Hoffman caught cold, and felt acute pains in his side. The pain increased, and Dr. Hoffman tried to cure it by injecting morphia. He did this with the syringe which he had used for injecting the glanders poison into the animals. Although it had been disinfected in glowing heat, some particles of the poison must have still been in it, for Dr. Hoffman grew worse, and his friends took him to the hospital. His colleagues were horrified when they saw him—the whole body being covered by terrible ulcers, which, when they were examined, proved to be filled with the glanders poison. After some days of suffering Dr. Hoffman died, adding another to the martyrs of science.

---

On October 17th, an influential meeting was held at Stafford, the Earl of Harrowby presiding, to decide whether a new hospital should be erected in the town, or the old hospital repaired. In opening the proceedings, the noble Chairman pointed out that the present institution had existed for 120 years. No doubt the difficulties which the institution had experienced in the past were caused by the growth of similar institutions in other localities, and which they were very pleased to see. But that fact did not lessen the requirements for a new hospital in Stafford as was shown from the fact that last year as many as 700 were treated at the infirmary; and this year, up to the present time, 551 had been treated. To show what a boon the institution was, his lordship pointed out that last year 300 were cured at the institution, and the remainder were more or less benefited. For the sum of £10,000, a model hospital could be built, providing accommodation for 80 persons—40 men, 30 women, and 10 children. It was recommended that the sum of £5,000 be raised by public subscriptions, which, together with what they would be able to realise for the old building, would be sufficient to justify them in starting with a building which should be capable further enlargement if it was found to be needed. The alternative was that they must struggle on, wasting several hundred pounds per year. Mr. T. Salt, M.P., moved that the work for the erection of a new hospital be proceeded with. Subsequent resolutions were passed as to the number of patients to be accommodated, and a representative committee was elected to carry the resolutions into effect.

